# Use of COX Inhibitors in Plastic Surgery Fibroproliferative Disorders: A Systematic Review

**DOI:** 10.3390/jpm15060257

**Published:** 2025-06-17

**Authors:** Yu Ting Tay, Elisha Purcell, Ishith Seth, Gianluca Marcaccini, Warren M. Rozen

**Affiliations:** 1 Department of Plastic and Reconstructive Surgery, Eastern Health, Melbourne, VIC 3128, Australia; 2 Faculty of Medicine, Peninsula Clinical School, Monash University, Melbourne, VIC 3199, Australia; 3 Department of Plastic and Reconstructive Surgery, Peninsula Health, Melbourne, VIC 3199, Australia; 4 Department of Plastic and Reconstructive Surgery, University of Siena, 53100 Siena, Italy

**Keywords:** COX inhibitors, fibroproliferative disorders, plastic surgery, systematic review, personalised medicine, precision medicine, phenotype-guided therapy

## Abstract

**Background/Objectives:** Fibroproliferative disorders (FPDs), such as Dupuytren’s contracture, scleroderma, capsular contracture, rhinophyma, and keloid scars, are characterised by excessive fibroblast activity and collagen deposition. These conditions are frequently encountered in plastic and reconstructive surgery and remain therapeutically challenging. Cyclooxygenase (COX) inhibitors have emerged as a potential adjunct therapy to modulate fibrotic pathways and improve clinical outcomes. This systematic review aims to evaluate the efficacy and safety profile of COX inhibitors in the management of plastic-surgery-related FPDs. In doing so, it explores how phenotype-guided and route-specific COX-inhibitor use may contribute to precision, patient-centred care. **Methods:** To identify eligible studies, a comprehensive search was conducted in MEDLINE, Embase, and the Cochrane Library. Data were synthesised using both tabular summaries and narrative analysis. The certainty of evidence was appraised according to the GRADE guidelines. **Results:** Thirteen studies from 1984 to 2024 met inclusion criteria, addressing FPDs such as hypertrophic scarring, Dupuytren’s contracture, and desmoid tumours, representing 491 patients. Of those, five studies were related to Dupuytren contracture, three studies were related to hypertrophic scar, and one study each was on topics related to scleroderma, keloid scar, osteogenesis imperfecta, actinic keloidalis nuchae/dissecting cellulitis of the scalp, and desmoid tumours. Nine studies reported clinical improvements (four demonstrating statistically significant outcomes), three showed no difference, and one did not assess outcomes. The thirteen studies show minor side effects from oral and topical COX inhibitors. The overall certainty of evidence was graded as “low.” **Conclusions:** COX inhibitors demonstrate promising efficacy with minimal adverse effects in the management of plastic-surgery-related FPDs. Their accessibility, safety, and potential to reduce fibrosis underscore the need for future high-quality, large-scale studies to establish definitive clinical recommendations.

## 1. Introduction

Fibroproliferative disorders (FPDs) are characterised by excessive production of connective tissue, leading to the degeneration of the standard structure and function of affected tissues [1]. With more than one-third of deaths in developed countries being accounted to a fibroproliferative disease, FPDs constitute a significant contributor to global morbidity and mortality [2,3]. FPDs include a diverse array of diseases and affect all body systems, ranging from systemic sclerosis to hepatic cirrhosis [2,3]. This review defines plastic-surgery-related FPDs as those managed or consulted on by plastic surgeons. In plastic and reconstructive surgery, FPDs encountered include Dupuytren’s contracture (DC), scleroderma, capsular contracture, rhinophyma, and keloid scars [4,5,6]. Despite the variation in clinical presentations of FPDs, the underlying mechanism of excessive fibroblast activity and collagen disposition underpins all FPDs [1,7]. Of important note, knowledge surrounding the pathogenesis of FPDs continues to emerge, and gaps in our understanding of FPDs continue to be identified [2,8,9,10]. Thus, the significant prevalence of FPDs in conjunction with the complexities of managing FPDs has created a pressing need to develop a safe and efficacious antifibrotic therapy.

The current management of FPDs is multifaceted and varies depending on the specific disorder. Further to this, the complex pathology of FPDs is challenging to manage as there are currently no approved treatments that directly target the mechanisms of fibrosis [2]. Developing successful antifibrotic treatments has been a significant challenge because fibrotic tissue comprises a densely cross-linked matrix and is relatively acellular [11]. Also, as fibrosis is an end-stage process that develops over multiple years, patients often present with late-stage fibrotic tissue [11]. Subsequently, for many FPDs, there are limited effective treatment modalities, and surgical interventions are usually the standard of care [7,12]. For example, when considering the management of symptomatic Dupuytren’s contracture, management is mainly surgical correction through procedures such as percutaneous needle fasciotomy and fasciectomy [12]. However, the complications of contracture recurrence, digital nerve injury, flexor tendon injury, haematoma, and infection remain common concerns associated with surgical operations [13,14]. Thus, with multiple end-stage FPDs being managed by plastic surgeons, we must research how FPDs can be improved. As disease behaviour and treatment response vary markedly between individuals, a personalised medicine framework that stratifies patients by fibroproliferative phenotype and predicted COX inhibitor sensitivity is increasingly advocated.

One potential emerging therapy to aid in managing FPDs is the use of cyclooxygenase (COX) inhibitors. Inhibitors of cyclooxygenases, specifically COX-1 and COX-2, include several anti-inflammatory drugs that are indicated for managing pain and inflammation in many disorders [15,16]. Over the past century, COX inhibitors have been extensively researched, which has resulted in many non-selective and selective COX-1 and COX-2 inhibitors being developed [16,17]. Recently, COX inhibitors have been proposed as a potential therapy to improve the outcomes of individuals with FPDs [18,19]. This notion is based on the growing body of evidence that suggests COXs play a role in fibroproliferation and the pathogenesis of abnormal wound healing [18,20,21]. Moreover, there is increasing evidence demonstrating COX inhibition with antifibrotic mechanisms across a range of FPDs [22,23,24]. With the benefits of safety, affordability, and accessibility, the use of COX inhibitors as an adjunct in the management of FPDs within plastic surgery may be a simple yet effective way to improve the outcomes of patients with FPDs.

This systematic review investigates COX inhibitors’ safety and efficacy in managing FPDs within plastic surgery. Specifically, we aimed to compare the effectiveness of COX-1 and COX-2 inhibitors in managing FPDs in plastic surgery to the current standard of care, that is, no use of COX inhibitors or other treatments.

## 2. Materials and Methods

This systematic review followed the Preferred Reporting Items for Systematic Reviews and Meta-Analyses (PRISMA) guidelines [25]. The protocol for this systematic review was prospectively registered on the International Prospective Register of Systematic Reviews (PROSPERO, registration ID CRD42025640450).

### 2.1. Selection Criteria

Studies eligible for this review included randomised controlled trials, cohort studies, case–control studies, and case series. Reviews, protocols, case reports, conference abstracts, and studies unable to be accessed in English were excluded. The type of participants eligible for this review were human patients with fibroproliferative disorders in plastic surgery. Examples include Dupuytren’s contracture, hypertrophic scarring, and localised scleroderma, which affects digits. Preclinical studies limited to in vitro and in vivo models were excluded. Studies investigating the intervention of cyclooxygenase (COX) inhibitors, specifically COX-1, COX-2, or non-selective COX inhibitors, were eligible. Examples include naproxen, aspirin, diclofenac, celecoxib, and sodium salicylate. Finally, studies using a comparator arm of no intervention or an alternative treatment were eligible. Examples of alternative treatment comparator arms included corticosteroids and laser therapy.

### 2.2. Primary and Secondary Outcomes

The primary outcome of this review was to systematically investigate the efficacy of COX inhibitors in managing FPDs in plastic surgery. The secondary outcome was to systematically investigate the safety profile of COX inhibitors in managing FPDs in plastic surgery.

### 2.3. Search Strategy

MEDLINE, Embase, and Cochrane Library databases were searched on 22 November 2024. Search terms such as ‘Adult’, ‘Surgery, Plastic’, ‘Keloid’, ‘Dupuytren’s Contracture’, and ‘COX inhibitors’ were combined using Boolean operators. The entire search strategy is detailed in Table A1. All publications from 1946 to 18 November 2024 were included. No language or other filter restrictions were applied to the search strategy. Further citation searching was conducted by reviewing references of relevant reviews and included studies.

### 2.4. Study Selection

All articles identified from the search strategy were imported into the Covidence Systematic Review Software (Extraction 1 tool). Duplicate articles were removed automatically and manually where applicable. Two review authors independently performed title and abstract screening (EP and YT) and full-text screening using the predefined selection criteria. In the case where full-text articles were unable to be retrieved, corresponding authors were contacted two times. After each screening stage, a third review author (IS) resolved any conflicts between review authors.

### 2.5. Data Collection

Relevant data were extracted from the included studies independently by review authors (EP and YT). As recommended by *The Cochrane Handbook*, the process of standard data extraction was followed [26]. This included extracting study characteristics (author, year, country, study type), sample size and target population, intervention details (type of COX inhibitor, dose, duration of treatment), and key outcomes (efficacy and safety). In any reviewer disagreement, a third review author was consulted to reach a consensus. When relevant data were missing from eligible studies, corresponding authors were contacted a total of two times. In line with a precision medicine perspective, we also extracted delivery route, dose, and patient-level characteristics (age, phenotype, comorbidities) to explore differential treatment effects.

### 2.6. Data Synthesis

Data were synthesised following a narrative review, combining tables and written results. Due to the limited number of studies and the conceptual heterogeneous nature between studies—specifically, differences in study populations, type of COX inhibitors used, comparator arms, and outcomes assessed—a meta-analysis and subgroup analysis were not appropriate to perform in this systematic review.

### 2.7. Quality Assessment

The risk of bias in each study was assessed independently by two review authors using the Cochrane Risk of Bias Tool for randomised trials and the ROBINS-I tool for non-randomised studies [27,28,29], the Newcastle-Ottawa Quality Assessment Scale for cohort studies, and the JBI critical appraisal tool for case series by two authors. Where differences in judgement of bias could not be resolved between two review authors, a third review author was consulted. The certainty of evidence was assessed using the Grading of Recommendations Assessment, Development and Evaluation (GRADE) [30].

## 3. Results

### 3.1. Search Results

**Figure 1:**
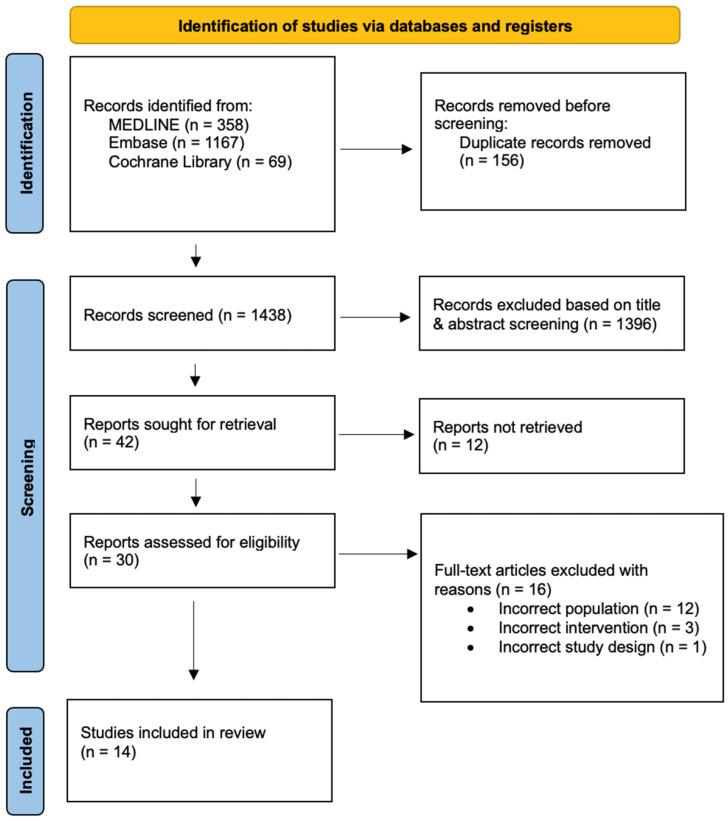
Preferred Reporting Items for Systematic Review and Meta-Analyses (PRISMA) flowchart and checklist.

A PRISMA flow chart summarising the screening process is presented in Figure 1. In applying the search strategy, a total of 1594 citations were identified. After removing 156 duplicated citations, 1438 unique records underwent title and abstract screening. Based on predefined selection criteria, 1396 records were excluded during title and abstract screening. A total of 42 studies underwent full-text screening, and 16 were excluded due to incorrect population (*n* = 12), incorrect intervention (*n* = 3), or incorrect study design (*n* = 1). A total of 13 studies were included in this systematic review through the screening process [31,32,33,34,35,36,37,38,39,40,41,42,43].

### 3.2. Characteristics of Included Studies

The characteristics of the included studies are summarised in Table 1. From the 13 included studies, there were 4 double-blinded randomised controlled trials (RCTs), 2 randomised controlled trials, 2 retrospective cohort studies, 1 prospective cohort study, and 4 case series. The most common FPDs investigated by these studies were Dupuytren’s contracture (*n* = 5), hypertrophic scarring (*n* = 3), and scleroderma with skin involvement (*n* = 1). A total of 491 patients were included in the studies extracted.

### 3.3. Primary Outcomes

#### 3.3.1. Ledderhose Disease

A case series by Boussaid et al. described two Tunisian brothers (17 and 14 years old) with Osteogenesis imperfecta and Ledderhose disease stemming from a first-degree consanguineous marriage. The two individuals were treated with NSAIDs, but there was no documentation on efficacy and conduction of further follow-up [39].

#### 3.3.2. Scleroderma

In a randomised controlled study by Beckett et al., 28 patients received dipyridamole (225 mg/day) and aspirin (975 mg/day). This study reported no significant difference between the COX inhibitor therapy and placebo groups. Symptoms such as skin induration, joint involvement, finger ulcers, and Raynaud’s phenomenon did not improve or worsen significantly in either group [31].

#### 3.3.3. Hypertrophic/Keloid Scars

A case series of three patients by Eker et al. examined the effects of infiltrating diclofenac sodium (50 mg) and 0.5% lidocaine (10 mL saline) into scar tissue [41]. In case 1, treatment resulted in decreased stiffness, tension, and contraction, as well as improved pain and burning sensations in lower limb scars. In case 2, the same injection combination of diclofenac sodium and 0.5% lidocaine helped alleviate phantom limb pain in a post-amputation lower limb scar. Finally, case 3 reported that the use of NSAIDs was associated with reduced pain, improved finger movement, and a reduction in scar size and tension in a post-carpal tunnel surgery scar after 1.5 years. Due to this study being a case series, no formal statistical analyses were performed on these reported measures [35].

In a more recent study by Opatha et al., Asiatic-acid-entrapped transfersomal gel (AATG) significantly reduced the melanin index (MI) at 2, 4, and 8 weeks by 6.40%, 12.49%, and 18.59% (*p* < 0.05), respectively [33]. In contrast, the placebo gel showed increases in MI of 3.37%, 4.69%, and 2.11% (*p* > 0.05). AATG also improved skin elasticity by 48.94%, 38.19%, and 44.36% (*p* < 0.05) at 2, 4, and 8 weeks. Skin surface hydration increased slightly by 6.24% and 5.59% at 4 and 8 weeks, compared to only a 0.87% increase at week 8 in the placebo group, though these results were not statistically significant (*p* > 0.05) [42].

#### 3.3.4. Postoperative Swelling

Husby et al. observed no significant differences in postoperative swelling following Dupuytren contracture release (*n* = 35) between the paracetamol (*n* = 12), naproxen (*n* = 12), and placebo (*n* = 11) groups. Mean postoperative swelling in the percentage of preoperative volume from paracetamol, naproxen, and placebo groups was 6.9%, 5.6%, and 8.2%, respectively, with a standard deviation of 3.7%, 3.8%, and 5.1%, respectively [32].

#### 3.3.5. Postoperative Pain

Vandepitte et al. conducted a RCT involving 32 subjects who underwent Dupuytren contracture release with collagenase Clostridium histolyticum. Patients received either bupivacaine HCl or bupivacaine liposome injection. All patients followed a standardised multimodal pain regimen, including acetaminophen, diclofenac, and tramadol for breakthrough pain. Notably, only three of the 32 patients required tramadol for breakthrough pain: one from the bupivacaine HCl group and two from the liposome bupivacaine group. One of the key conclusions from this study was that the majority of patients (13 of 16) who received liposome bupivacaine injections did not require additional lidocaine injections, unlike the comparator group who received bupivacaine hydrochloride alone [36].

In Husby et al., no patient who received naproxen (*n* = 12) following Dupuytren contracture release required additional analgesia. This is compared to two patients in the placebo group and no patients in the paracetamol group requiring additional analgesia. Importantly, this result was not statistically significant, and a small sample size further limited the study [32].

In another study by Noto et al., NSAIDs such as loxoprofen and celecoxib were used to control pain after collagenase Clostridium histolyticum injections for Dupuytren contracture [37]. Out of a total of 41 patients receiving collagenase injections, 14 patients received NSAIDs. Pain was reported as being improved dramatically in all 41 patients within 7 days of collagenase injection, and no data were compared between those who used NSAIDs and those who did not [37].

Van Nuffel et al. investigated the use of celecoxib (200 mg daily) versus placebo after collagenase injection for Dupuytren contracture release [43]. The key finding from this study was that patients in the celecoxib group reported lower pain scores and higher patient satisfaction compared to those in the placebo group. The Total Passive Extension Deficit also increased in the celecoxib group [43].

Wong et al. observed no significant difference in the onset of moderate to severe postoperative pain between patients receiving an intraoperative opioid regimen and those with an opioid-sparing regimen (such as propofol, acetaminophen, ketorolac, and ketamine). Notably, the study found that a higher number of adjuvants, such as acetaminophen combined with ketorolac, resulted in a decrease in moderate to severe postoperative pain [34].

#### 3.3.6. Acne Keloidalis Nuchae (AKN) and Dissecting Cellulitis of the Scalp (DCS)

A case series by Michelen-Gomez et al. involving three DCS patients and one AKN patient treated with 1% diclofenac gel showed improved pain symptoms. The three DCS patients had a reduction in papules and visible hair growth, while the AKN patient showed fewer non-tender erythematous papules on the occipital scalp and neck after 1 month [41].

#### 3.3.7. Desmoid Tumour

Robles et al.’s case series included a case of a 7-year-old patient with a desmoid tumour being treated with sorafenib (200 mg daily) and celecoxib (100 mg twice daily). After 14 months of therapy, MRI scans showed tumour stabilisation, decreased pain, improved range of motion, and a softer mass under the skin [38].

### 3.4. Secondary Outcomes-Safety Profile of COX Inhibitors in FPD

The 13 studies reviewed reported minor side effects from the oral and topical use of COX inhibitors, including dipyridamole, aspirin, acetylsalicylic acid, celecoxib, and acetaminophen. In Beckett et al.’s study, one patient on dipyridamole (225 mg/day) and aspirin (975 mg/day) developed aggravated oesophageal stricture [31]. In the case series by Husby et al., one patient using naproxen after Dupuytren contracture surgery reported stomach pain/regurgitation, and another experienced bloating [32]. Furthermore, in Van Nuffel et al.’s study, two patients in the celecoxib group reported stomach pain, with one patient discontinuing the medication after 6 weeks. Minor events such as shoulder tendinitis, hand and foot pain, rhinitis, and a broken tooth were also reported, but these were deemed unrelated to the medication [43]. In Noland et al.’s study, among 182 patients taking acetylsalicylic acid, there were 18 with skin tears compared to 21 of 187 in the non-anticoagulated group and 3 of 50 on other anticoagulation. One tendon rupture was reported in the acetylsalicylic acid group. Lymphadenopathy occurred in 5 of 182 acetylsalicylic acid patients, 2 of 8 on apixaban, and 11 of 187 without anticoagulants [40]. The only side effect reported for topical COX inhibitors was mild burning upon applying 1% diclofenac gel in DCS patients (Michelen-Gomez et al.) [41]. No adverse effects were noted in patients using Asiatic-acid-entrapped transfersomal gel (AATG); specifically, no skin reactions were reported during the irritation test [42].

### 3.5. Quality Assessment

**Figure 2:**
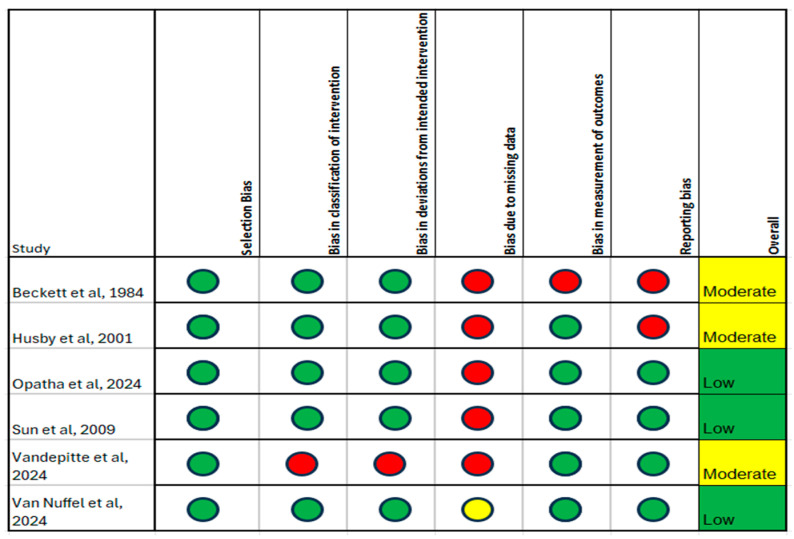
Revised Cochrane risk-of-bias tool for randomised trials (RoB 2) showing 3 studies with moderate [31,32,36] and 3 studies with low [33,42,43] overall risk of bias. Color code: Green = low risk; Yellow = moderate risk; Red = high risk of bias.

**Figure 3:**
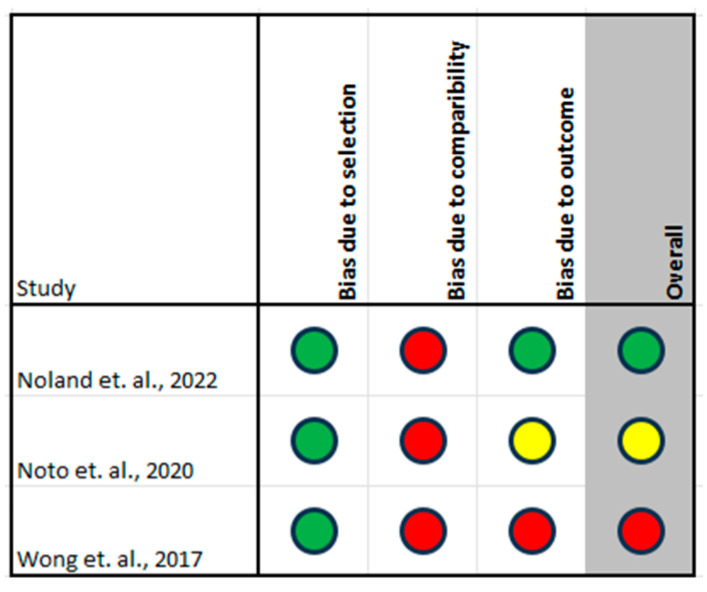
Newcastle-Ottawa Quality Assessment Scale for cohort studies showing one low, one moderate, and one high overall risk of bias [34,37,40]. Color code: Green = low risk; Yellow = moderate risk; Red = high risk of bias.

**Figure 4:**
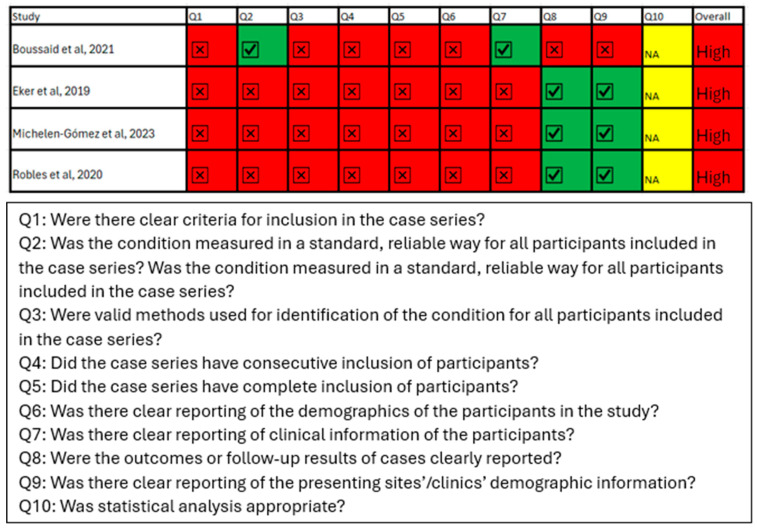
JBI critical appraisal tool for case series. Showing all four case series have high overall risk of bias [35,38,39,41]. Symbols: ✓ = criterion fulfilled; ✗ = criterion not fulfilled; NA = not applicable. Color code: Red = High risk of bias, Yellow = Moderate risk, Green = Low risk; Grey = Not applicable.

The risk of bias assessment of included studies is summarised in Figure 2, Figure 3 and Figure 4. In using the risk of bias tool for randomised studies, three studies were assessed as overall low risk of bias, specifically Opatha et al., Sun et al., and Van Nuffel et al., and three studies were judged as having a moderate risk of bias, specifically Beckett et al., Husby et al., and Vandepitte et al. [31,32,33,36,42,43]. The Newcastle-Ottawa scale was applied to assess the risk of bias in the cohort studies, all of which were judged to have an overall moderate risk of bias [34,37,40]. Finally, the JBI tool was used to assess the risk of bias in case series, of which three studies were judged as having an overall high risk of bias, namely Boussaid et al., Michelen-Gomez et al., and Robles et al., while the Eker et al. study was judged as having an overall moderate risk of bias [35,38,39,41].

Following the GRADE approach for assessing the certainty of evidence in systematic reviews, randomised trials were initially rated as having high-quality evidence, and observational studies were initially rated as having low-quality evidence [39]. The randomised control trials and observation studies were both downgraded due to imprecision. No other factors were present to downgrade or upgrade the certainty of evidence. Thus, this overall certainty of evidence was deemed moderate for randomised controlled trials and very low for observational studies included in this review.

## 4. Discussion

Aberrant tissue remodelling, driven by a series of inflammatory, proliferative, and fibrotic events in response to injury, can affect multiple organs and is collectively referred to as FPDs [1]. These disorders are broadly categorised into aggressive and benign types. Aggressive FPDs typically involve the accumulation of mesenchymal cells and their connective tissue products in critical anatomical locations, as seen in idiopathic pulmonary fibrosis, hepatic cirrhosis, myelofibrosis, and systemic sclerosis [44]. In contrast, benign FPDs are characterised by excessive extracellular matrix deposition, leading to loss of compliance, impaired function, and slow contractures [1]. Within plastic and reconstructive surgery, the most common examples include Dupuytren’s contracture, hypertrophic scars, and keloids [1].

The potential of COX inhibitors to manage plastic-surgery-related FDPs, such as hypertrophic and keloid scars, Dupuytren’s contracture, Ledderhose disease, and dissecting scalp cellulitis, appears promising. The studies in this review explore three main COX inhibitor treatment modalities for the management of plastic-surgery-related FPDs: intralesional injection, topical therapy, and oral medication.

A study involving intralesional injections of 100 mg diclofenac and 50 mg of 0.5% lidocaine in 10 mL saline showed positive outcomes for postoperative painful scars [35]. Injection of COX inhibitors shows potential in treating FPDs like hypertrophic scar, but higher-quality research with a bigger sample size is needed to confirm this [35].

Looking into topical COX inhibitors for the management of FPDs, AATG demonstrated promising results; however, specific methods, dosages, and patient numbers in the placebo and treatment groups were not disclosed. Meanwhile, diclofenac sodium gel aids in managing conditions such as acne keloidalis nuchae and dissecting cellulitis of the scalp [41,42]. In a study by Sun et al., 1 out of 36 patients in the control group developed a keloid, indicating the potential for COX inhibitors to reduce keloid formation. However, more high-quality studies are needed to confirm this [33].

Studies investigating COX inhibitors for plastic-surgery-related postoperative swelling, such as that by Husby et al., suggest that more patients are needed to assess statistical significance. However, early indications suggest that naproxen may outperform placebo administration. Interestingly, this use of naproxen for postoperative swelling is also being investigated using a wide range of other non-related FPD surgeries, for example, hand, joint, and oral surgery [32,44,45,46].

COX inhibitors are widely used in pain management across various plastic-surgery-related FPDs. Five studies reviewed indicated the efficacy of COX inhibitors administered orally. For instance, in Husby et al.’s study, patients who did not receive COX inhibitors required additional analgesics. Noto et al. reported that 14 of 41 patients received either loxoprofen or celecoxib for pain management, with all patients experiencing significant pain relief after 7 days of collagenase Clostridium histolyticum injections for Dupuytren’s contracture [32]. However, a direct comparison between groups was not made. Vandepitte’s study administered a multimodal analgesic regimen of acetaminophen and diclofenac, providing reasonable pain control with minimal breakthrough pain [36]. Other studies (e.g., Vannuffel and Wong et al.) suggest that COX inhibitors may reduce postoperative pain following procedures such as carbon dioxide laser therapy used in hypertrophic scar management and collagenase injection in DC [34,43].

Furthermore, this review also identified studies investigating the use of COX inhibitors for less well-known plastic-surgery-related FPDs. Of note, a case series suggested that combining celecoxib with sorafenib may help control desmoid tumours in paediatric patients [38]. Another study found that the COX inhibitors dipyridamole and aspirin did not significantly improve finger-related symptoms in scleroderma [31]. Finally, another study investigated Ledderhose disease, generally treated conservatively with NSAIDs. However, this paper had no follow-up data on efficacy and did not provide specific medication recommendations [39].

In regard to the safety profile of COX inhibitors for plastic-surgery-related FPDs, COX inhibitors have been widely used to manage inflammation and pain. The most commonly reported side effects include nausea, vomiting, stomach pain, heartburn, and altered bowel habits [15]. Within this systematic review, the reported side effects are consistent with those well documented in current literature. For example, one patient using antiplatelet therapy for scleroderma disease experienced exacerbated oesophageal symptoms, which may be attributable to disease progression rather than medication [31]. Husby et al. also noted one case of constipation in the placebo group, and two other patients required additional analgesics (morphine and paracetamol/codeine) [32]. It appears that in Husby et al.’s article, both the placebo and the COX inhibitor group experience gastrointestinal-related side effects, which may not be related to the intervention [32]. Van Nuffel et al.’s article highlights the potential risk of stomach pain associated with the use of celecoxib compared to the placebo group, as none of the patients in the placebo group experienced symptoms, while two patients in the celecoxib group exhibited similar symptoms [43]. Only one out of four patients experienced mild burning from the application of diclofenac gel on draining lesions, but that is likely due to open wounds rather than side effects of the treatment. Moreover, the patient proceeded to use this treatment for at least seven more months without any documented issues [31]. In the Noland et al. study, although it has been reported that acetylsalicylic acid has the highest number of skin tears, the group has more participants compared to other groups. Therefore, caution must be exercised when considering this as a potential side effect [40]. In many studies, the side effects were not documented, including Bossaid et al., Ekar et al., Noto et al., Vandepitte et al., and Sun et al. [33,35,36,37,39]. In summary, based on the widely studied COX inhibitors from previous research and the reported side effects in this systematic review, the use of COX inhibitors appears safe in plastic-surgery-related FPDs.

Although established definitions for FPDs exist, this study identified a lack of a precise framework addressing plastic-surgery-related fibroproliferative conditions. Plastic surgery units routinely manage conditions such as Dupuytren’s contracture, plantar fibromatosis (Ledderhose disease), keloid scars, and hypertrophic scars. However, conditions, such as systemic sclerosis and CREST syndrome (calcinosis, Raynaud’s phenomenon, oesophageal dysmotility, sclerodactyly, and telangiectasia), are typically managed by rheumatologists, with referrals to plastic surgery primarily for finger involvement. Thus, the heterogeneity between plastic-surgery-related FPDs is a key limitation of this study.

In addition, the studies included in the review assessed the efficacy of COX inhibitors in plastic-surgery-related FPDs, which broadly varied across the included studies. For instance, the case series by Bossaid et al. explored Ledderhose disease in the context of osteogenesis imperfecta, while studies by Noto et al. and Vandepitte et al. investigated pain intensity following collagenase Clostridium histolyticum injections for Dupuytren’s contracture [36,37,39]. Another limitation is that some articles did not include COX inhibitor dosages [34,39]. Boussaid et al. and Noland et al. did not mention the specific medication dose and period [39,40]. Ekar et al.’s article has three cases, all of which have improvement, but they did not mention the suggested dose [35]. Furthermore, some studies produce convincing data on the efficacy of COX inhibitors in treating plastic-surgery-related FDPs, but have a limited number of patients to produce statistically significant evidence [31,32,38,42]. Similarly, in Michelne-Gomez et al.’s study, all four patients have varying amounts and frequency in use and combination with varying other therapies [41]. The 41 patients in Noto et al. have varying doses and frequencies of lexoprofen and celecoxib [37]. Some of the studies also do not have any comparison with other interventions such as placebo. For instance, it is difficult to determine whether using celecoxib helps control desmoid tumours or if sorafenib helps with it in Robles et al.’s article [38]. There is also no comparison in the case series investigating the efficacy of 1% diclofenac sodium gel in individuals with dissecting cellulitis of scalp and acne keloidalis nuchae [41].

Taken together, our findings suggest that COX inhibitor effectiveness is modulated by fibroproliferative subtype, administration route, and dosage, echoing the core principles of personalised medicine. This review recommends further research to delineate COX inhibitors’ efficacy and safety profile in plastic-surgery-related FPDs. Studies of high-quality designs and patients with larger sample sizes will provide crucial evidence on the efficacy of COX inhibitors in this patient population. To improve the quality of studies and thus the certainty of proof, we recommend that future studies pre-register the study protocol, incorporate standardised outcomes to measure efficacy in the given disorders, and report methodology in greater detail, specifically the use of blinding and method of randomisation. Prospective biomarker-enriched trials are now needed to predict responders, refine dosing, and minimise adverse events on an individual basis. With additional high-quality studies, we can then incorporate the use of COX inhibitors in plastic-surgery-related disorders.

## 5. Conclusions

COX inhibitors, available in oral, topical, and injectable forms, hold significant potential in the management of plastic-surgery-related fibroproliferative diseases, including scleroderma, Ledderhose disease, hypertrophic and keloid scars, postoperative swelling, procedural pain, AKN/DCS, and desmoid tumours. The studies reviewed suggest minimal side effects and positive outcomes; however, the quality of the evidence remains low. Given the wide accessibility and clinical use of COX inhibitors, further high-quality research is essential to establish their safety and efficacy in these contexts. Although current evidence is low to moderate in availability, it supports integrating COX inhibitors into personalised treatment algorithms that match drug, dose, and delivery route to the patient’s specific fibroproliferative phenotype.

## Figures and Tables

**Table 1:** Characteristics of included studies.

Study	Study Design	Population	Intervention	Comparator	Outcomes	Limitation	Conclusions	Side Effect
Beckett et al., 1984 [31]	Double-blind RCT	Individuals (all ages) with scleroderma skin involvement and at least two other systems (*n* = 41)	Dipyridamole 75 mg and aspirin 325 mg TDS orally for 12 or 24 months.	Placebo	Correlation of clinical involvement and laboratory findings: platelet survival, fibrinogen, plasma renin activity, biomechanical score, finger/arm systolic BP ratio. Clinical parameters: change in skin induration, joint findings, number of finger ulcers, severity of Raynaud’s phenomenon, myopathy, oesophageal changes, pulmonary findings, renal insufficiency.	Limited number of subjects included.	Significant correlation (*p* < 0.01) appeared between biochemical test scores of hand function and involvement of skin, joint, and multiple organs, and finger/arm ratio of systolic BP and number of finger ulcers. No patients showed significant improvement. Some patients showed improvement in 1–3 parameters, though simultaneous worsening in other parameters.	One patient from aspirin group has aggravated oesophageal symptoms.
Husby et al., 2001 [32]	Double-blind RCT	Individuals (all ages) undergoing DC release or CTS release (*n* = 35, CTS not included)	Naproxen 500 mg BD vs. paracetamol 1000 mg QID given 3 days post-operatively.	Placebo	Swelling measurement and use of supplementary analgesia (opioid).	Limited number of subjects included.	DC release: naproxen observed to be mildly better than paracetamol and placebo, though this was not statistically significant. Power of study limited by number of patients. Use of naproxen after operation does not require rescue analgesia, 2 patients in placebo group required rescue analgesia post-DC. Naproxen may be a useful analgesia in the acute postoperative phase. 1 patient from the placebo group developed a keloid scar.	One patient using naproxen after Dupuytren contracture surgery reported stomach pain/regurgitation.
Sun et al., 2009 [33]	RCT	Individuals (all ages) undergoing major plastic surgery under general anaesthesia (120 patients), but only one (*n* = 1) had plastic-surgery-related FPDs	Postoperative celecoxib 400 mg immediately after surgery, followed by 200 mg BD for 3 days postoperatively. Perioperative group received 400 mg celecoxib 30–90 min before surgery, 400 mg placebo immediately after surgery, and 200 mg celecoxib BD for 3 days postoperatively.	Placebo	VRS pain score, surgical/wound complications, and patient satisfaction.	This paper does not look into plastic-surgery-related FPDs. However, was included as one patient in the control group developed keloid scar.	Keloid was formed in 1 patient in the control group (*n* = 36).	One patient has deep vein thrombosis.
Wong et al., 2017 [34]	Retrospective cohort	Paediatric patients with hypertrophic scar receiving laser rehabilitation (*n* = 88)	Intraoperative opioid regime (morphine, fentanyl, or morphine + fentanyl) + adjuvant (acetaminophen and ketorolac). Dosage of medications not reported.	Opioid-sparing regime	Face Legs Activity Cry Consolability (FLACC) scale or on the 0 to 10 scale (pain).	Did not look into COX inhibitor alone vs. placebo.	No statistically difference in onset of moderate to severe pain between groups.	Not mentioned.
Eker et al., 2019 [35]	Case series	Adults with hypertrophic scar tissue and neuropathic pain (*n* = 3)	All cases use diclofenac sodium 100 mg and lidocaine SC injection 50 mg 0.5% of varying frequency: Case 1—three repeats with one-week intervals. Case 2—two repeats with a one-week interval. Case 3—four repeats with one-week intervals.	Nil	Case 1: Stiffness of scar determined by palpation. Patient’s pain and burning sensation. Case 2: Patient’s pain and phantom sensation. Case 3: Pain score (NRS), limitation of finger movement, size of scar tissue, and tension.	Variable in dosage and follow-up between patients.	All 3 cases resulted in an improvement in measured outcomes.	Not mentioned.
Vandepitte et al., 2019 [36]	RCT	Dupuytren contracture release with collagenase (*n* = 32)	Liposome bupivacaine 1.33% 5 mL + 2.5 mL bupivacaine hydrochloride 0.5% per nerve (n = 16).	Bupivacaine hydrochloride 0.5% 7.5 mL alone per nerve (n = 16).	Requirement of further analgesia for manipulation and pain score through 3 days after treatment.	The main objective of this paper is to investigate other treatment. However, this is included as all of the subjects took combination of oral analgesia including COX inhibitor.	Additional liposome bupivacaine prolonged sensory block and pain score. All subjects received the multimodal regimen for pain control: acetaminophen, diclofenac, and tramadol.	Only side effects not specific to COX inhibitors are reported (bleeding wound, dizziness, headache, itching of skin, nausea, sleepless nights).
Noto et al., 2020 [37]	Prospective cohort study	Individuals (all ages) receiving collagenase *Clostridium histolyticum* injection for DC (*n* = 41)	NSAID (lexoprofen and celecoxib) self-administered up to 7 days post-DC release (varying dose and frequency of use).	Nil comparator (though 27 out of 41 used no NSAIDs, but study did not report data comparing between the two groups).	Pain (visual analogue scale, VAS) and revised version of the Short-Form MacGill Pain Questionnaire (SF-MPQ-2).	The main objective of this study does not look into efficacy of NSAIDs.	Average reported peak pain was at 9 h, most did not use NSAIDs at that time. NSAIDs may have been used after the extension procedure. Pain after collagenase *Clostridium histolyticum* injection was controlled by minimal use of NSAIDs.	Not mentioned.
Robles et al., 2020 [38]	Case series	Paediatric patients with desmoid tumour (*n* = 1); case 1 excluded due to intra abdominal tumour	Case 2—sorafenib 200 mg and celecoxib 100 mg BD for 14 months.	Nil	Tumour size, pain, range of motion, and functionality.	Only one patient can be included in the analysis.	Tumour size markedly decreased, substantial decrease in pain, improved right upper limb range of motion and functionality.	No side effects for both patients.
Boussaid et al., 2021 [39]	Case series	17- and 14-year-olds with Plantar fibromatosis and Osteogenesis imperfecta (*n* = 2)	Oral NSAIDs (specific medication, dose, and period not reported).	Nil	No follow-up after patient intervention.	It just describes the use of COX inhibitor, did not have outcome.	Not reported.	Not mentioned.
Noland et al., 2022 [40]	Retrospective cohort	Individuals (all ages) receiving collagenase injections for DC (*n* = 197)	Aspirin (dose not reported).	Warfarin (dose not reported) or no anticoagulation	Complications: skin tear, tendon rupture, lymphadenopathy.	Looks into complications of various anticoagulation, no efficacy of treatment mentioned.	8 skin tears occurred in the aspirin group, 3 in the warfarin group, and 11 in the non-anticoagulated group (no significant difference). 1 tendon rupture in the aspirin group. 11 lymphadenopathy in the non-anticoagulated group, 5 in the aspirin group, and 2 in the apixaban group. No significant difference in complications.	As per conclusion.
Michelen-Gómez et al., 2023 [41]	Case series	Individuals (all ages) with dissecting cellulitis of scalp (DCS) and acne keloidalis nuchae (AKN) (*n* = 4)	1% diclofenac sodium gel (varying amounts and frequencies in use) and in combination with varying other therapies.	Nil	Patient 1–3: pain score (NRS) and clinical examination (lesion and hair growth). Patient 4: pain score (NRS) and clinical examination (lesion).	Variable dosage and follow-up for each patient, limited number of patients.	Patient 1: DCS improvement at 1 and 3 months. Patient 2: DCS improvement at 3 months. Patient 3: DCS improvement at 3 months. Patient 4: AKN improvement at 1 month.	Mild burning upon applying 1% diclofenac gel.
Opatha et al., 2024 [42]	Double-blind RCT	Healthy Thai adults (age 20–40) with hypertrophic scars (*n* = 14)	Asiatic-acid-entrapped transfersomal gel (AATG) 0.25 g to affected scar region BD for 8 weeks.	Placebo	Melanin index (MI), skin elasticity (measured with Cutometer), skin surface hydration (measured with Corneometer)	Limited number of subjects included.	AATG significantly decreased the MI at weeks 2, 4, 8 (6.40%, 12.49%, 18.59%, *p* < 0.05). The placebo gel had a slight increase in MI (3.37%, 4.69%, 2.13%, *p* > 0.05). AATG resulted in more skin elasticity compared to placebo gel. Skin surface hydration increased with AATG at weeks 4 and 8 (6.24%, 5.59%, *p* > 0.05). The placebo gel showed a mild increase at week 8 (0.87%, *p* > 0.05). AATG resulted in a significant decrease in MI and net increase in skin elasticity at 8 weeks.	No side effects.
Van Nuffel 2024 [43]	Double-blind RCT	Collagenase injection for DC (*n* = 32)	Celecoxib 200 mg once daily for 12 weeks.	Placebo	Total Passive Extension Deficit (TPED)/ray, TPED of the individual near joints, Tubiana index, Disability of Arm, Shoulder and Hand score (DASH), and visual analogue scale (VAS) for pain and satisfaction.	Relatively low number of participants. VAS and DASH may not be sensitive enough to detect the differences.	Adjuvant peroral administration of celecoxib shows significantly greater improvement in TPED and metacarpal contracture (with beneficial effect up to 24 months), but less pronounced effect on interphalangeal joints. VAS for pain and satisfaction was better at 6 and 12 weeks in the celecoxib group. Other outcome parameters did not differ significantly between the two groups.	Two patients from celecoxib group reported stomach pain. Pain in hands and feet, rhinitis, and broken tooth in celecoxib group unlikely related to celecoxib.

Abbreviations: AATG, Asiatic-acid-entrapped transfersomal gel; AKN, acne keloidalis nuchae; BD, twice a day; BP, blood pressure; COX, cyclooxygenase; CTS, carpal tunnel syndrome; DASH, Disability of Arm, Shoulder and Hand score; DC, Duputreyn’s contracture; DCS, dissecting cellulitis of scalp; FLACC, Face Legs Activity Cry Consolability; FPDs, fibroproliferative diseases; MI, melanin index; NRS, numerical rating scale; NSAID, non-steroidal anti-inflammatory drugs; QID, four times a day; RCT, randomised controlled trial; SC, subcutaneous; TDS, three times a day; TPED, Total Passive Extension Deficit; VAS, visual analogue scale; VRS, verbal rating scale.

## Data Availability

All datasets are available upon request.

## Abbreviations

The following abbreviations are used in this manuscript:

AATG	Asiatic-acid-entrapped transfersomal gel
AKN	Acne keloidalis nuchae
BD	Twice a day
BP	Blood pressure
COX	Cyclooxygenase
CTS	Carpal tunnel syndrome
DASH	Disability of Arm, Shoulder and Hand score
DC	Dupuytren’s contracture
DCS	Dissecting cellulitis of scalp
FLACC	Face Legs Activity Cry Consolability
FPDs	Fibroproliferative disorders
GRADE	Grading of Recommendations Assessment, Development and Evaluation
MI	Melanin index
NRS	Numerical rating scale
NSAID	Non-steroidal anti-inflammatory drugs
PRISMA	Preferred Reporting Items for Systematic Reviews and Meta-Analyses
PROSPERO	International Prospective Register of Systematic Reviews
QID	Four times a day
RCT	Randomised controlled trial
SC	Subcutaneous
TDS	Three times a day
TPED	Total Passive Extension Deficit
VAS	Visual analogue scale
VRS	Verbal rating scale

## Appendix A

**Table A1:** Search strategy used for the systematic review, including MeSH terms and free-text keywords for population, condition, and intervention.

#	Query
1	Exp Humans/ or exp Adult/ or exp Child/ or exp Surgery, Plastic/
2	(human$ or adult$ or child$ or plastic surger$ or reconstructive surger$).tw.
3	1 or 2
4	Exp Keloid/ or exp Cicatrix, Hypertrophic/ or exp Dupuytren’s Contracture or exp Implant Capsular Contracture/ or exp Contracture/ or exp Rhinophyma/ or exp Scleroderma, localized/ or exp Scleroderma, systemic/
5	(keloid$ or cicatr$ or hypertrophic scar or dupuytren$ or contracture or rhinophyma$ or scleroderma$).tw.
6	4 or 5
7	Exp Cyclooxygenase Inhibitors/ or exp Anti-Inflammatory Agents, Non-Steroidal
8	(cyclooxygenase inhibitor$ or COX-1 inhibitor$ or COX-2 inhibitor$ or non-steroid$ or NSAID or prostaglandin-endoperoxide synthase inhibitor$ or indomethacin or naproxen or aspirin or piroxicam or tolmetin or meclofenamate or ketorolac or flurbiprofen or ketoprofen or ibuprofen or fenoprofen or sodium salicylate or diflunisal or sulindac or diclofenac or celecoxib or meloxicam or etodolac or etoricoxib or lumiracoxib).tw.
9	7 or 8
10	3 and 6 and 9
